# Herpesviruses and their genetic diversity in the blood virome of healthy individuals: effect of aging

**DOI:** 10.1186/s12979-022-00268-x

**Published:** 2022-03-12

**Authors:** Arttu Autio, Jalmari Kettunen, Tapio Nevalainen, Bryn Kimura, Mikko Hurme

**Affiliations:** 1 Faculty of Medicine and Health Technology, Tampere University, Arvo Ylpön katu 34, 33520 Tampere, Finland; 2 Gerontology Research Center (GEREC), Tampere, Finland; 3 Science Centre, Pirkanmaa Hospital District, Tampere, Finland

**Keywords:** Immunosenescence, Metatranscriptomic, Virome, Aging, Ageing, Epstein-Barr virus, EBV, Herpesviruses, RNA sequencing, RNA-seq

## Abstract

**Background:**

As we age, the functioning of the human immune system declines. The results of this are increases in morbidity and mortality associated with infectious diseases, cancer, cardiovascular disease, and neurodegenerative disease in elderly individuals, as well as a weakened vaccination response. The aging of the immune system is thought to affect and be affected by the human virome, the collection of all viruses present in an individual. Persistent viral infections, such as those caused by certain herpesviruses, can be present in an individual for long periods of time without any overt pathology, yet are associated with disease in states of compromised immune function. To better understand the effects on human health of such persistent viral infections, we must first understand how the human virome changes with age. We have now analyzed the composition of the whole blood virome of 317 individuals, 21–70 years old, using a metatranscriptomic approach. Use of RNA sequencing data allows for the unbiased detection of RNA viruses and active DNA viruses.

**Results:**

The data obtained showed that Epstein-Barr virus (EBV) was the most frequently expressed virus, with other detected viruses being herpes simplex virus 1, human cytomegalovirus, torque teno viruses, and papillomaviruses. Of the 317 studied blood samples, 68 (21%) had EBV expression, whereas the other detected viruses were only detected in at most 6 samples (2%). We therefore focused on EBV in our further analyses. Frequency of EBV detection, relative EBV RNA abundance and the genetic diversity of EBV was not significantly different between age groups (21–59 and 60–70 years old). No significant correlation was seen between EBV RNA abundance and age. Deconvolution analysis revealed a significant difference in proportions of activated dendritic cells, macrophages M1, and activated mast cells between EBV expression positive and negative individuals.

**Conclusions:**

As it is likely that the EBV RNA quantified in this work is derived from reactivation of the latent EBV virus, these data suggest that age does not affect the rate of reactivation nor the genetic landscape of EBV. These findings offer new insight on the genetic diversity of a persistent EBV infection in the long-term.

**Supplementary Information:**

The online version contains supplementary material available at 10.1186/s12979-022-00268-x.

## Background

As we age, the functioning of the human immune system declines. Resulting from this are increases in morbidity and mortality associated with infectious diseases, cancer, cardiovascular disease, and neurodegenerative disease in elderly individuals, as well as a weakened vaccination response [1]. These age-related changes to the human immune system are referred to as immunosenescence. The disproportionate number of deaths of elderly individuals in the ongoing COVID-19 pandemic has been a grim reminder of the susceptibility of older immune systems to novel pathogens [2]. Functionally, immunosenescence is associated with an increased rate and severity of infections, autoimmunity, and decreased response to vaccinations in elderly individuals [3]. At the cellular level, the hallmark of immunosenescence is the accumulation of the senescence-associated secretory phenotype of CD8 positive T cells, which have lost the CD28 antigen required as a co-stimulatory signal in T cell activation. In addition to this, the proportion of CD14 positive monocytes and macrophages is increased (associated with the general increase of inflammation, often called inflammaging) and the proportion of antibody-producing B cells is decreased [4, 5].

During the last decades, substantial evidence has accumulated demonstrating that the human body is colonized by microbial communities (bacteria, fungi, viruses, and protozoa), which have a clear impact on human health. The human virome, the collection of all viruses present in an individual, is not limited to disease states, as chronic but asymptomatic viral infections are thought to be common [6]. The word infection is used here only to denote the presence of exogenous viruses in the body, as the viruses may be silent and inactive, without any form of active infection. However, study of viromes is challenging, especially due to the small size of viral genomes and the high degree of sequence similarity between them [7]. Knowledge of the human virome remains limited [8].

Many of the viruses persistently residing in humans belong to the herpesvirus family, such as Epstein-Barr virus (EBV), cytomegalovirus (CMV) and herpes simplex 1 (HSV-1), and their impact on human health may be much greater than what is currently understood about infections that do not typically have severe pathology [5]. For example, cytomegalovirus (CMV) is thought to add to the progressive accumulation of senescent dysfunctional T-cells, contributing to the frailty syndrome and mortality [9]. While EBV has been thought to have similar impact to immunosenescence as CMV [10], the connections between EBV, immunosenescence and disease are not fully clear [5]. It is important to note that herpesvirus infections do have the potential to be severe, which usually occurs in conditions of immune immaturity, age-associated immune decline or immune dysregulation [11]. Herpesviruses establish persistent infections that are occasionally reactivated [5]. In case of persistent, latent CMV infection, monocyte differentiation results in the transcription of CMV genes without reactivation [12]. Identification of detrimental immunomodulatory elements of the microbiome is needed to better understand how the immune system ages and what could be done to slow its decline.

It seems likely that the various defense mechanisms of the body, both adaptive and innate immune mechanisms, would have a role in the modulation of the composition of the microbiomes in the various locations of the body. This should be clear e.g. in the case of the blood virome, i.e. the “antigens” are in close contact with the cells of the immune system. There are several reports about the composition of the virome in different body compartments, though the results vary between studies [13, 14]. In the case of blood virome, Moustafa et al. [15] demonstrated that 94 different DNA viruses were detectable, however, many of those were due to widespread DNA contamination of commercial reagents.

One aspect of the virome that remains woefully under-explored is the diversity of viral species and subspecies in human populations as well as within individual human hosts. Viral diversity is multifaceted, as it can be studied across hosts or within-hosts, it may change over time, and it exists on different levels such as species, strain and nucleotide level. The same individual human can be infected with multiple different strains of the same virus simultaneously [16], while separate copies of viral genomes can have small, nucleotide level differences between copies. Within-host viral populations may evolve towards greater diversity for the sake of increasing readiness to adapt to new selective pressures [17]. Yet diversity may also be reduced over time as the more robust variants of the virus become increasingly dominant [18]. One example of the impact of viral diversity is seen with the COVID-19 variants and the significant differences seen between them in infectivity [19].

In the present study we have used RNA sequencing (RNA-seq) data from the Genotype-Tissue Expression (GTEx) project that has been obtained from blood samples taken from individuals of various ages. To fully understand the behavior and impacts of a virus, one must know how an infection develops in individuals over time and how the virus behaves on a population level. Our aim was therefore to investigate age-associated differences in the human virome by identifying viruses, studying their relative viral RNA abundance as well as viral diversity. Use of RNA sequencing data allows us to study RNA viruses as well as active DNA viruses.

## Results

RNA-seq data obtained from blood samples was analyzed to identify viral RNA. On average, sample data consisted of 51.5 million raw read pairs (minimum 38.8 million, maximum 316.6 million). After quality control, the samples had 26.6 million quality read pairs on average (min 0.5 million, max 55.4 million). As only unambiguous mapping was accepted, the mean alignment rate to human genome was 79.3% (min 54.8%, max 88.7%). After human read subtraction, 2.3% of non-human reads (min 0.5%, max 7.7%) aligned to non-viral microbiome genomes on average.

A total of 12 different virus species were observed among the 317 samples. Of these, 87 samples contained at least one virus species. Among the observed virus species, the Epstein-Barr virus was the most prevalent, identified in 68 individuals. Other prevalent viruses were herpes simplex virus 1 (HSV-1) and cytomegalovirus (CMV), that were observed in 5 and 6 individuals, respectively. Rare occurrences (3 or less positive individuals) included human mastadenovirus C, variety of papillomaviruses, Torque Teno viruses and betacoronavirus (Table 1).

**Table 1:** Summary of the viruses detected in the analysed blood samples (*N* = 317)

Species	Virus subtype of reference sequence	GenBank accession of reference sequence	Sequence positive samples	Mean sequence abundance in species positive samples	Number of species positive samples	Mean species abundance in species positive samples
Epstein-Barr virus	HKNPC1 (EBV type 1)	JQ009376	63	0.659	68	1.414
	M81	KF373730	49	0.421		
	IM-3	MK973061	32	0.219		
	HN4	AB850649	19	0.076		
	NKTCL-SG05	MH144216	3	0.016		
	Akata (EBV type 1)	KC207813	3	0.012		
	variant BZLF1-C (EBV type 1)	KF826537	2	0.007		
	undefined (LMP mRNA)	M58153	1	0.005		
Herpes simplex virus 1	MacIntyre	MN136523	5	3.588	5	22.200
	F	GU734771	5	3.532		
	isolate HSV-v29_day1_culture2	MG708287	5	1.740		
	F-13	MH999842	5	1.537		
	KOS, variant Kinchington	JQ780693	5	1.535		
	RDH193	KT425108	5	1.533		
	unknown (dbp/pol genes)	X03181	5	1.387		
	McKrae	JQ730035	5	1.258		
	CM1	KX791792	5	1.226		
	K86	MH999839	5	0.964		
	isolate HSV-v29_day-90_culture1	MG708286	5	0.907		
	isolate ZW6	KX424525	5	0.571		
	M-19	MH999850	5	0.541		
	17	NC_001806	5	0.474		
	isolate 1319_2005	LT594108	5	0.396		
	isolate HSV-v29_site12_day3	MG708289	4	0.245		
	K47	MH999838	3	0.236		
	OD4	JN420342	3	0.205		
	McKrae, clone contig00012	KX791997	2	0.165		
	F-18 g	MH999847	2	0.091		
	isolate B^3 × 1.5	KU310661	1	0.035		
	isolate B^3 × 1.3	KU310659	1	0.035		
Human cytomegalovirus	AD169	FJ527563	5	0.249	6	0.647
	Towne	LT907985	4	0.367		
	U11	GU179290	1	0.031		
Human mastadenovirus C	serotype 57	HQ003817	3	0.419	3	1.230
	serotype 6, isolate Tonsil 99	HQ413315	2	0.398		
	serotype 1, strain SH2016	MH183293	2	0.346		
	serotype 2	MF315029	1	0.066		
Torque teno virus 13	isolate TCHN-A	AF345526	3	0.848	3	0.848
Betapapillomavirus 1	serotype 195, isolate ACS380	KR816182	2	0.438	3	0.616
	serotype 98	FM955837	1	0.178		
Betacoronavirus 1	HCoV_OC43/Seattle/USA/SC9430/2018	MN306053	2	0.331	2	0.331
Torque teno virus 29	isolate TTVyon-KC009	AB038621	2	0.310	2	0.310
Betapapillomavirus 4	isolate Beta04_TVMGc2024	MF588686	1	0.960	1	0.960
Gammapapillomavirus 1	serotype 4	NC_001457	1	0.896	1	0.896
Gammapapillomavirus 9	isolate Gamma09_w27c39c	MF588712	1	0.426	1	0.426
Betapapillomavirus 2	serotype 23	U31781	1	0.190	2	0.337
	serotype 107	EF422221	1	0.147		

The detected viruses are identified by species name, subtype name of the reference sequence as well as GenBank accession of the reference sequence. The number of samples positive for a specific virus is shown on both species and subtype level. Mean RNA abundance is similarly shown on both species and subtype level.

To evaluate the association between age and viral species, each sample was classified as young or old, using the cut-off age of 60 years. Only in the case of EBV was the number of species-positive samples high enough to allow group comparison. With aforementioned age cut-off, the number of EBV positive samples in young and old groups were 43 and 25, respectively. Frequencies of EBV positive persons were not significantly different between age groups (two-sided Pearson’s chi-square test, *p* = 0.33) (Table 2). Total EBV RNA abundance in each sample was estimated by summing the abundances of all EBV reference sequences. The mean total EBV RNA abundance was 1.585 and 1.119 reads per million quality reads in young and old individuals, respectively. No significant difference in abundance was observed between groups (non-parametric Mann-Whitney U-test, *p* = 0.16) (Table 2).

**Table 2:** Epstein-Barr virus positive persons and mean of total RNA abundance by age group

Age group	Number of persons	Number of EBV positive persons	Mean of total EBV RNA abundance in positive persons
Age < 60	216	43	1.585
Age ≥ 60	101	25	1.119

Difference in frequencies of EBV positive persons was not significant (two-sided Pearson’s chi-square test, *p* = 0.33). Difference in means was not significant (non-parametric Mann-Whitney U-test, *p* = 0.16). Total EBV RNA abundance is shown as reads per million quality reads.

Potential linear age-associated differences in EBV RNA abundance were additionally investigated. No significant correlation was seen between EBV RNA abundance and donor age in EBV positive samples (Spearman’s rank correlation coefficient: −0.13, *p*-value: 0.29).

As differences in aging and in virus infection have been reported between the sexes, potential differences in samples from female and male sample donors were investigated (Table 3). There were 200 male and 117 female sample donors. The number of EBV positive samples was 39 from male sample donors and 29 from female sample donors. Based on this, 19.5% of samples from male individuals and 24.8% of samples from female individuals exhibited EBV expression. Frequencies of the EBV positive persons were not significantly different between the sexes (two-sided Pearson’s chi-square test, *p* = 0.27). The mean total EBV RNA abundance was 1.454 and 1.360 reads per million quality reads for male and female individuals, respectively. No significant difference in abundance was observed between the sexes (non-parametric Mann-Whitney U-test, *p* = 0.64). Furthermore, no age-associated significant differences were seen in abundance when each sex was tested separately, as non-parametric Mann-Whitney U-test resulted in a *p*-value of 0.26 for male sample donors and 0.41 for female sample donors. No significant correlation was seen between EBV RNA abundance and age for men (Spearman’s rank correlation coefficient: −0.16, *p*-value: 0.33) or for women (Spearman’s rank correlation coefficient: −0.09, *p*-value: 0.63), when tested separately.

**Table 3:** Epstein-Barr virus positive persons and mean of total RNA abundance by sex

Sex	Number of persons	Number of EBV positive persons	Mean of total EBV RNA abundance in positive persons
Male	200	39	1.454
Female	117	29	1.360

Difference in frequencies of EBV positive persons was not significant (two-sided Pearson’s chi-square test, *p* = 0.27). Difference in means was not significant (non-parametric Mann-Whitney U-test, *p* = 0.64). Total EBV RNA abundance is shown as reads per million quality reads

To investigate potential relationships between EBV and proportions of different immune cells, deconvolution analysis was used to estimate the proportion of different immune cell types from the studied bulk RNA-Seq data. Results from the digital cytometry tool CIBERSORTx showed a significant *p*-value (*p* ﻿≤ 0.05) for 215 of the 317 samples. A significant *p*-value from CIBERSORTx indicates that the results of the deconvolution are significantly different from results that would have been obtained by random chance. Only these 215 samples with high deconvolution performance were utilized in downstream analyses. Of the 68 samples that had EBV expression, 43 had a significant CIBERSORTx *p*-value. The cell proportions seen in these 43 samples were compared to the 172 samples that did not show EBV expression and had significant deconvolution fitting accuracy. Of the 22 different immune cell types differentiated in the CIBERSORTx LM22 data, significant differences (*p* ﻿≤ 0.05) in cell proportions between EBV expression positive and negative individuals were seen with the cell types: macrophages M1, activated dendritic cells, and activated mast cells (Table 4). When the 22 immune cell types were pooled into larger groups (lymphocytes, T cells, T cells CD8, T cells CD4, B cells, NK cells), no significant differences were seen.

**Table 4:** Differences in the proportions of immune cell types between EBV expression positive and negative samples

Cell type	EBV pos median %	EBV neg median %	***p***-value
B cells, naive	5.73	5.04	0.971
B cells, memory	0.00	0.00	0.287
Plasma cells	3.41	2.88	0.185
T cells, CD8	2.41	2.34	0.985
T cells, CD4 naive	4.57	4.44	0.823
T cells, CD4 memory resting	5.01	7.48	0.389
T cells, CD4 memory activated	3.65	2.24	0.096
T cells, follicular helper	0.00	0.00	0.419
T cells, regulatory	0.00	0.15	0.291
T cells, gamma delta	1.08	0.00	0.432
NK cells, resting	7.17	7.79	0.354
NK cells, activated	0.00	0.00	0.620
Monocytes	9.79	6.23	0.235
Macrophages, M0	3.49	2.42	0.422
**Macrophages, M1**	**0.00**	**0.53**	**0.043**
Macrophages, M2	0.00	0.00	0.987
Dendritic cells, resting	0.96	0.78	0.696
**Dendritic cells, activated**	**2.91**	**1.67**	**0.004**
Mast cells, resting	3.01	1.04	0.187
**Mast cells, activated**	**0.00**	**0.10**	**0.007**
Eosinophils	0.75	0.74	0.848
Neutrophils	6.52	5.04	0.256

Each of the 22 functionally defined human hematopoietic cell subsets included in the CIBERSORTx LM22 data were tested using non-parametric Mann-Whitney U-test. Of the 215 samples for which CIBERSORTx provided a high confidence deconvolution result, 43 samples had EBV expression compared to the 172 samples that did not. CIBERSORTx results are given as relative proportions of the 22 cell types and the median values for EBV expression positive and negative samples for each cell type are shown in this table as percentages. The cell types with significant *p*-values are shown in bold

**Fig. 1:**
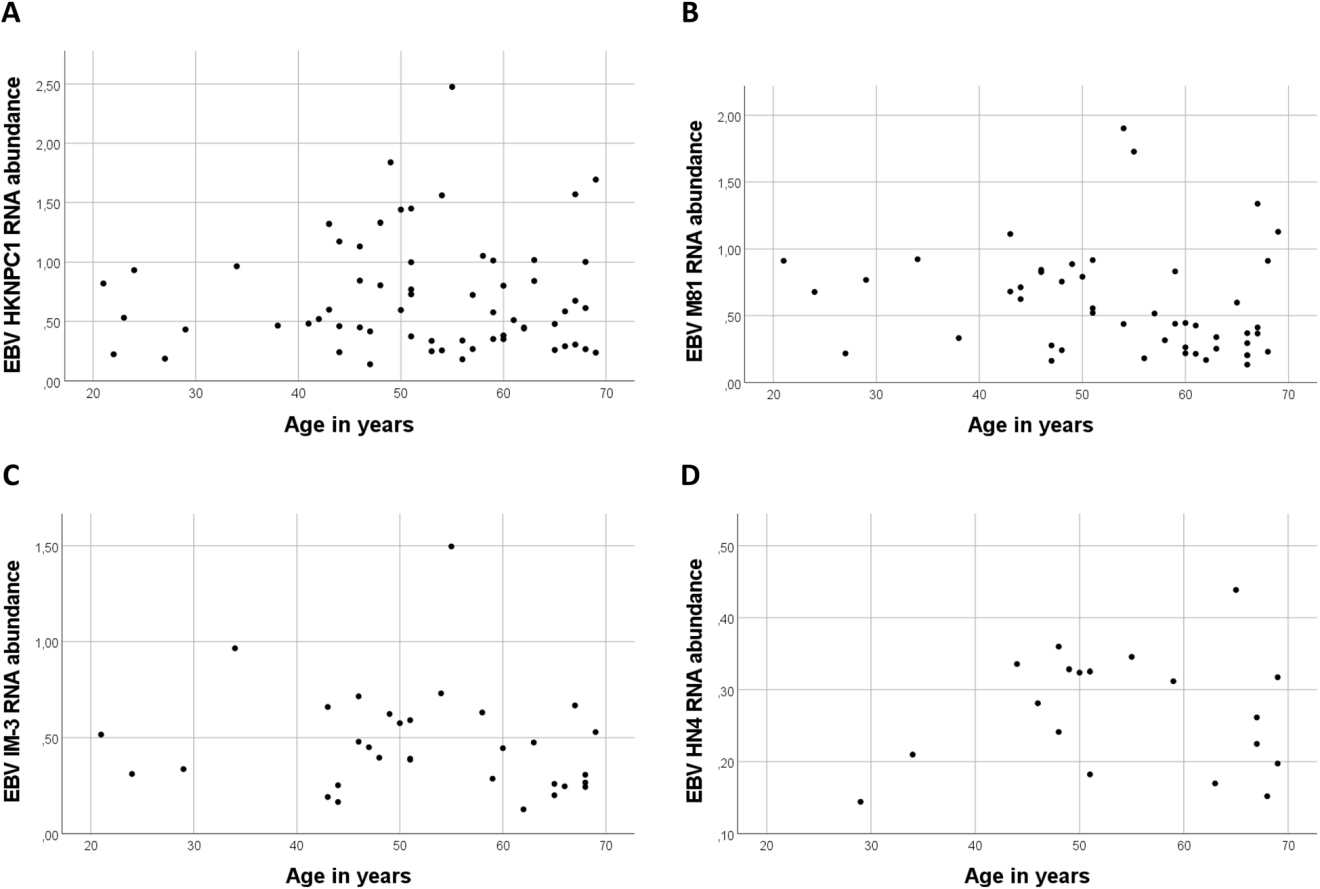
Sample-wise RNA abundances of the four most prevalent EBV reference sequences with age. No clear association is seen with sample donor age and reference sequence EBV RNA abundance

**Fig. 2:**
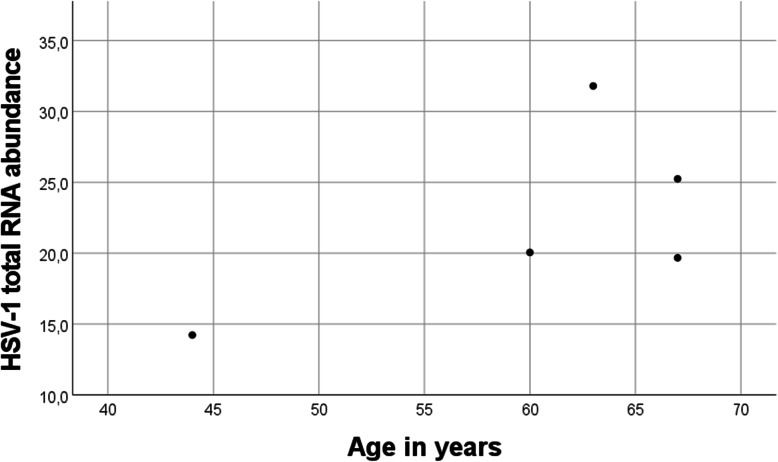
Sample-wise total RNA abundances of HSV-1. Number of individuals where HSV-1 RNA was observed, was 5. Of these, four were considered old and one was young

For several virus species, such as EBV and HSV-1, multiple reference sequences were detected. For EBV, the alignment to 8 reference sequences was observed. Of these, four were relatively prevalent: reference sequences HKNPC1, M81, IM-3, and HN4 were observed in 63, 49, 32, and 19 individuals, respectively. Figure 1 shows the observed RNA abundances of these four reference sequences in relation to individuals’ age. In the case of HSV-1, there were 5 individuals where presence of RNA was confirmed, and 22 reference sequences. Majority of sequences were observed in all 5 individuals and total HSV-1 abundance was high in these persons (Table 1, Fig. 2).

**Fig. 3:**
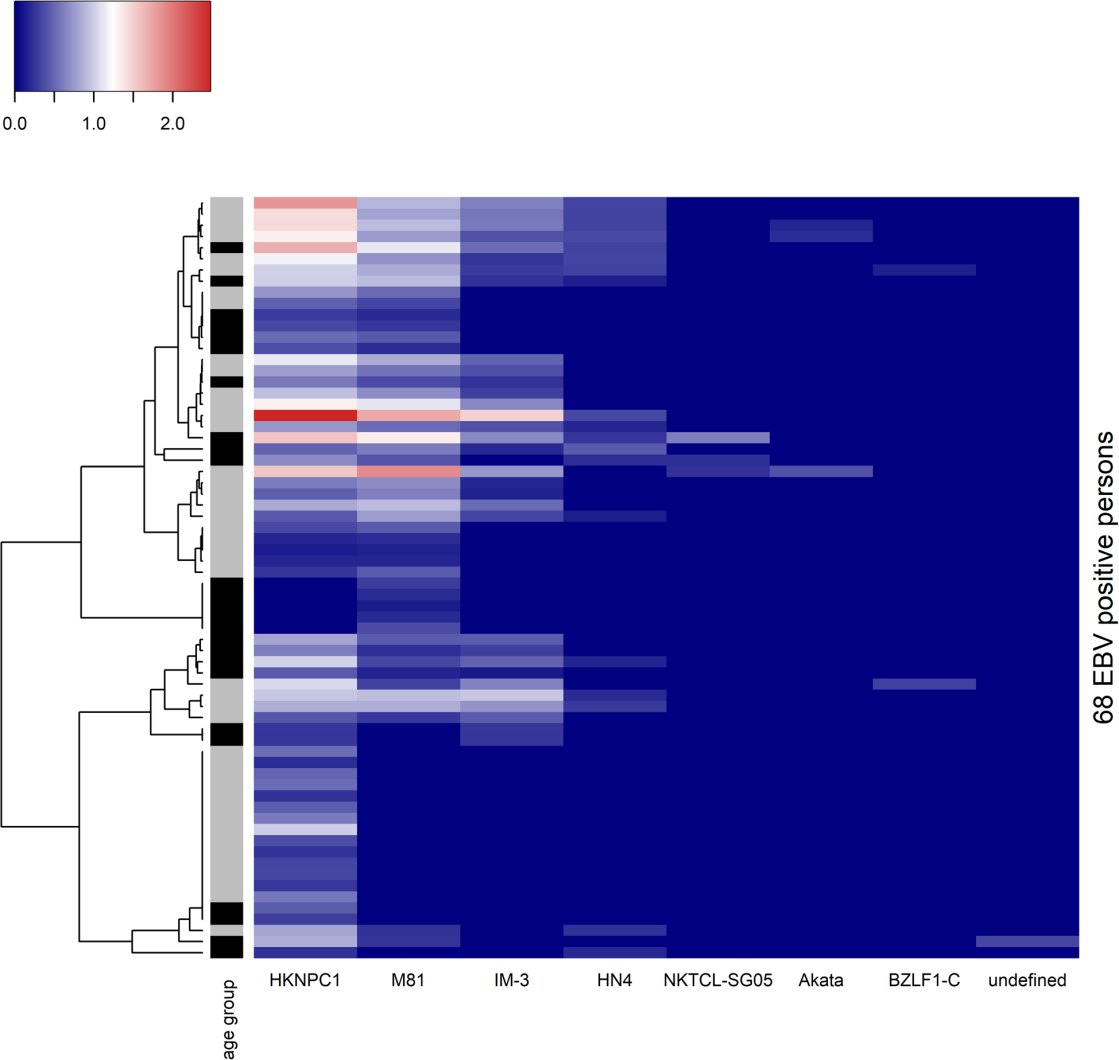
RNA abundance of different EBV reference sequences in EBV positive persons. Colour scale from blue to red indicates low to high abundance. Subtype of GenBank accession ID is used as name for each EBV reference sequence on x-axis. Persons were clustered according to Euclidean distance measure of abundance values (dendrogram on the left). Age group of each person is indicated by side bar (grey: age < 60, black: age ≥ 60)

To assess the age-associated difference in viral diversity, the presence and variety of observed reference sequences in young and old individuals were considered. Only in the case of EBV was there considerable variation in the prevalence of observed sequences. Figure 3 shows that EBV positive subjects were not grouped according to their age group when clustered by their EBV abundance profile. To confirm this, multistep-multiscale bootstrap resampling was done on the EBV abundance profiles to quantify the uncertainty involved in the clustering. No significant clustering, as defined by *p*-value ≤ 0.05, was seen along age group lines, nor was there significant clustering by sex. No significant age-associated clustering was seen when male or female individuals were clustered separately.

## Discussion

The results indicate that Epstein-Barr virus (EBV) was the most frequently expressed virus in the studied samples. Of the 317 studied blood samples, 68 (21%) had EBV expression, whereas the other viruses were only detected in at most 6 samples (2%). Therefore, for most of the viruses detected in this study, with the exception of EBV, the frequency that they appear in the studied samples was too low to be able to make meaningful statistical comparisons between age groups. We therefore focused on EBV in our further analyses. Frequency of EBV detection, relative EBV RNA abundance and the genetic diversity of EBV was not significantly different between age groups (21–59 and 60–70 years old). Neither was a significant correlation seen between EBV RNA abundance and age of sample donor. This lack of significant difference between age groups and absence of significant correlation with age was true even when testing separately for male and female sample donors.

The RNA-seq data used in this work measures frequency and magnitude of EBV reactivation rather than seroprevalence of EBV, as seroprevalence of EBV is likely to be very high in the individuals studied in this work (21–70 years old). EBV seroprevalence has been reported to be as high as 89% already in 18–19 year olds [20], meaning that seroprevalence of EBV between young adults and the elderly does not differ significantly. In this context, the results indicate that aging does not contribute to EBV reactivation.

EBV is known to maintain specific gene expression in latency. Latency-encoded genes include several nuclear antigens (EBNA), membrane proteins (LMP1, LMP2A, and LMP2B), and non-coding RNAs (EBER) [21]. Furthermore, EBV is known to reactivate in stressful conditions [22], and the general reactivation frequency seems to be quite high [23]. We observed RNA widely from EBV genome outside of the aforementioned latent genes, thus implying that this RNA expression results from EBV reactivation. With this interpretation, there was ongoing reactivation event in 21% of our samples. Moreover, according to some studies, EBV reactivation is not simply an on and off event, but rather there possibly exists partial micro-reactivation states, where some subset of reactivation genes is expressed [5].

It has been shown that genomic diversity of EBV increases during acute EBV infection, which is then followed by convergence as the infection is resolved and latency is established [18]. It has also been suggested that during aging, immune systems control over latent EBV is decreased, allowing EBV to establish chronic infectious state through reactivation [10]. In this context, we hypothesized that chronic infection would result in increased genomic diversity as seen in the acute infection. However, there was no significant age-associated difference in the EBV diversity, which implies that aged state differs from that of acute EBV infection. It is possible that our sample population was too young to reveal the age-associated chronic infectious state. It is also possible that nucleotide-level comparison using DNA sequencing would still indicate smaller scale differences, such as in a study by Weiss et al. [18], in which nucleotide-level EBV diversity was seen to decrease over time in the same individuals in favor of a more robust variant.

The cell type proportion deconvolution analysis showed that of the 22 functionally defined human hematopoietic cell subsets in CIBERSORTx LM22 data, significant differences in cell proportions between EBV expression positive and negative individuals were seen with the cell types: macrophages M1 (non-parametric Mann-Whitney U-test, *p* = 0.043), activated dendritic cells (*p* = 0.004), and activated mast cells (*p* = 0.007). Macrophages M1 and activated mast cells were present in significantly greater proportions in EBV expression negative samples. Activated dendritic cells were present in significantly greater proportions in EBV expression positive samples. Both the strongest significance and the greatest relative difference in proportions between EBV expression positive and negative samples was seen with activated dendritic cells (Table 4). Activation of dendritic cells in connection to EBV has been previously reported [24]. No significant differences were seen when these more specific cell types were pooled into larger groups (lymphocytes, T cells, T cells CD8, T cells CD4, B cells, NK cells), indicating that the observed significant differences are specific to the aforementioned three cell types.

Overall, the viruses detected in this study corresponded well with an earlier study conducted with GTEx data, although frequencies of viruses were generally lower in our results [13]. This was probably due to acceptance of only unambiguous read alignments which enabled study of viral diversity. Our results were dominated by DNA viruses, such as herpesviruses, and this is a common result from earlier blood virome studies [25]. Still, non-transcribing DNA viruses may remain undetected with RNA-seq. In addition, certain RNA viruses may have been missed because of polyA enrichment protocol [26]. Further, sensitivity of virus detection was probably suboptimal also because no viral enrichment was done. On the other hand, this approach avoids many types of bias in frequency and abundance of detected viruses [27].

As expected from Kumata et al., anellovirus transcription seemed rare in this study. Anelloviruses are single-stranded DNA viruses of family *Anelloviridae* whose viral DNA load have been associated with immunosenescence [28]. Although the blood of the majority of healthy people is anellovirus positive by PCR [29], studies using RNA-seq give conflicting results on whether it is commonly transcribed in healthy blood [13, 25]. These differences may result from geography or its relatively low titer in blood [30] as high-throughput sequencing has lower sensitivity than PCR [31]. Many virome studies have detected bacteriophages and other non-human viruses from healthy human blood [15, 32]. However, the scope of this study was on well-established human viruses and the virome pipeline was performed accordingly.

It is worth noting that the 5 HSV-1 positive persons had diverse HSV-1 transcripts and that 4 of them were old individuals. HSV-1 is another herpesvirus which establishes latent infection for life in majority of people. Its reactivation, sometimes asymptomatic, is believed to contribute to immunosenescence [33] although the exact reactivation mechanism is unknown [34]. Multiple variants in the same individual have been reported [35, 36]. Here, a small number of HSV-1 positive samples made statistical comparisons infeasible, yet this is something of which further study would be warranted.

Due to the curated and clustered nature of the Virosaurus90 reference sequences used in this work, the viral genes present in the data were analysed to verify the presence of viral diversity. When EBV alignments were analysed, our read data was found to cover genes that were common among the detected EBV reference sequences. Because reference sequences of the same virus species had common gene homologs, high confidence read alignment to multiple of them suggests viral diversity, even when the reference database consists of only representative sequences. The viral genes and their respective read counts can be found in Additional file 1.

## Conclusions

This metatranscriptomic study of the viromes of 317 individuals of varying ages found EBV to be by far the most commonly expressed virus. The frequency of EBV detection, relative EBV RNA abundance and the genetic diversity of EBV was found to not be significantly different between age groups (21–59 and 60–70 years old). No significant correlation was seen between EBV RNA abundances and age. No significant differences were seen between the sexes, nor were there age-associated differences when tested separately for male and female sample donors. As it is likely that this EBV is derived from reactivation of the latent virus, these data suggest that age does not significantly affect the rate of reactivation nor the genetic landscape of EBV.

## Methods

### Origin of raw data

The polyA-enriched RNA-sequencing data studied in this work originates from non-diseased whole blood samples taken as part of the Genotype-Tissue Expression (GTEx) Project (dbGaP accession number phs000424.v8.p2). The GTEx project as a whole is an ongoing effort to build a comprehensive public resource to study tissue-specific gene expression and regulation. As part of the project, 17,382 samples have been collected from organ and tissue donors, originating from 54 types of tissue and from 948 individuals. Samples used in the project are collected from non-diseased tissue sites and are studied using primarily molecular assays, including WGS, WES, and RNA-Seq. The whole blood samples studied in this work originate from 317 persons. Each person contributed one sample and their age varied between 21 and 70 years. All donors were surgical patients or post-mortem donors [37]. For whole blood collection the GTEx Tissue Harvesting Work Instruction states that the collection site preference is the femoral vein, while the subclavian vein and heart are other possible sites [38]. The Instruction also states that the preference of location will vary for organ donors (usually arterial line for beating heart donors) compared to non-beating heart tissue donors (venous route) [38]. Eligibility criteria and sequencing of biological samples has been described in more detail elsewhere [37, 38].

### Virus reference

Virosaurus is a curated virus genome database, aimed at facilitating clinical metagenomics analysis [39]. The viral reference sequences used in this work are from Virosaurus90, which consists of viral GenBank reference sequences clustered to 90% similarity. Representing each cluster in Virosaurus90 is a representative sequence chosen by selecting the longest sequence in the cluster. Due to the large genome size of herpesviruses and poxviruses, they are represented by shorter gene sequences in Virosaurus90 instead of full reference genomes. In this work, a “reference sequence” refers to the chosen representative GenBank reference sequence. Virus subtypes of representative reference sequences were retrieved from original publications via the GenBank database. Here, both accession ID and name of subtype are used to identify a virus reference sequence.

### Virome pipeline

A Bioinformatics pipeline modified from a study by Li et al. [25] was run in Puhti supercomputer cluster of CSC (Espoo, Finland). Paired-end RNA-sequencingreads of 317 samples were downloaded from Sequence Read Archive in FASTQ format with SRA Toolkit (v2.10.8). Low-quality ends (Phred score < 20) and Illumina Universal Adapters were trimmed with TrimGalore (v0.6.4; https://github.com/FelixKrueger/TrimGalore; 10.5.2021). Other quality filtering was performed with following qualifiers of PRINSEQ (lite v0.20.4) [40]: read length ≥ 50 nucleotides, mean quality score of read ≥25, proportion of ambiguous bases ≤1%, filter all kinds of duplicates, DUST score measuring low complexity ≤7. Quality filtering was confirmed with FastQC (v0.11.8; https://www.bioinformatics.babraham.ac.uk/projects/fastqc; 10.5.2021).

Quality reads were subtracted sequentially by aligning them with STAR (v2.7.1a) [41] against human reference genome (GCF_000001405.26_GRCh38_genomic.fna from NCBI) and non-viral Human Microbiome Project genomes (2236 archae, bacterial and fungi genomes downloaded 15.11.2019 from NCBI) [42]. Only uniquely mapping reads were subtracted (−-outFilterMultimapNmax 1). Remaining reads were aligned with Bowtie2 (v2.4.1) [43] against reference sequences of human viruses in Virosaurus90 database [39]. Only high confidence reads (MAPQ value ≥10) mapped to virus references were quantified with idxstats tool of SAMtools (v1.10) [44].

### Detailed analysis of viral abundance

If a virus reference sequence consisted of multiple genes in Virosaurus90 database, reads mapping to different genes were summed. After this, a virus reference sequence was considered detected in a sample if its total read count in the sample was ≥5 [25]. Virus reference sequences marked as unverified were removed from the results. In addition, read alignments were manually verified to be of viral origin by submitting covered reference regions to BLASTN search [45] against nt database of NCBI. This led to removal of certain viruses with high level of homology to human genes (HIV-1, HIV-2, enterovirus A). Then, read count of each virus sequence in each sample was normalized per million quality read pairs:

$$ viral\kern0.17em abundance=\frac{virus\kern0.17em read s\kern0.17em in\kern0.17em sample}{quality\kern0.17em read\kern0.17em pairs\kern0.17em in\kern0.17em sample}\times {10}^6 $$

Read count data was processed in RStudio (R version 3.6.1; https://www.r-project.org; 10.5.2021). Difference in means was tested with non-parametric Mann-Whitney U-test and difference in frequencies was tested with two-sided Pearson’s chi-squared test (IBM SPSS Statistics version 27). To support presence of viral diversity, GFF3 annotations for each GenBank reference genome of detected viruses were downloaded from NCBI Nucleotide database and compared to both Virosaurus90 database and aligned virus reads with the help of BEDTools (version 2.29.0) [46], custom Bash scripts and custom Python scripts. The heatmap and its clustering, based on Euclidean distance metric, were plotted with R package heatmap3 [47].

### Deconvolution analysis

Deconvolution analysis of different immune cell types was done utilizing the digital cytometry tool CIBERSORTx [48]. CIBERSORTx estimates the abundances of cell types in a mixed cell population, based on gene expression data and known connections between genes and cell types. CIBERSORTx provides an empirical *p*-value to evaluate deconvolution performance. The *p*-value is calculated by comparing the resulting cell type fractions with fractions that would have been obtained by random chance [49]. CIBERSORTx was run utilizing CIBERSORTx LM22 data, consisting of 22 functionally defined human hematopoietic subsets [50], as the signature matrix. Batch correction was enabled, and the number of permutations set to 1000 for significance analysis. TPM normalized gene expression values, from whole blood samples taken from the studied 317 individuals, were used as the mixture matrix.

### Hierarchical clustering of samples based on EBV expression

Hierarchical clustering of the samples based on EBV viral RNA abundance was performed to determine whether any statistically significant clustering along age group lines could be seen. Spearman correlation was used as the distance metric, which is robust against outliers and non-Gaussian distributions, and can capture nonlinear relationships [51, 52]. Ward’s minimum increase of sum-of-squares was used as the linkage method, which has been reported to perform better with RNA-seq expression data than the more traditional methods of average and complete linkage [51]. Multistep-multiscale bootstrap resampling was done with 10,000 bootstrap replications to evaluate the uncertainty involved in the clustering [53]. An approximately unbiased (AU) *p*-value is obtained, which indicates the bias corrected percentage of dendrogram variants where the specific cluster was observed.

## Supplementary Information

**Additional file 1.** Supplementary tables of virus genes. The file contains information on what viral genes are present in the reference sequences and the read counts attributed to each viral gene.

## Data Availability

The raw RNA-seq data of the GTEx project analyzed in this work can be accessed for research purposes through the database of Genotypes and Phenotypes (dbGaP) system. The dbGaP accession number for the project is phs000424.v8.p2. Access to GTEx protected data, which includes the raw sequencing data, requires an approved dbGaP application.

## Abbreviations

EBV: Epstein-Barr virus
CMV: Cytomegalovirus
HSV-1: Herpes simplex 1
RNA-seq: RNA sequencing
